# Supplementary material for: HIVprotI: an integrated web based platform for prediction and design of HIV proteins inhibitors
Source: J Cheminform. 2018 Mar 9;10:12. doi: 10.1186/s13321-018-0266-y (PMC5845081; doi:10.1186/s13321-018-0266-y)
Supplement: Supplementary file 2 — Additional file 2. Source code of HIVProtI web server. [file 13321_2018_266_MOESM2_ESM.zip › HIVprotI_Source-code/hivprotI/weka-3-6-6/doc/weka/gui/sql/package-summary.html]

weka.gui.sql


---


|  |  |  |
| --- | --- | --- |
| **Overview** \| **Package** \| Class \| **Tree** \| **Deprecated** \| **Index** \| **Help** \| **Weka's home** | |  |
| **PREV PACKAGE**   **NEXT PACKAGE** | **FRAMES**    **NO FRAMES**     **All Classes** |  |


---

## Package weka.gui.sql

| **Class Summary** | |
| --- | --- |
| **ConnectionPanel** | Enables the user to insert a database URL, plus user/password to connect to this database. |
| **DbUtils** | A little bit extended DatabaseUtils class. |
| **InfoPanel** | A simple panel for displaying information, e.g. |
| **InfoPanelCellRenderer** | A specialized renderer that takes care of JLabels in a JList. |
| **Messages** | Messages. |
| **QueryPanel** | Represents a panel for entering an SQL query. |
| **ResultPanel** | Represents a panel for displaying the results of a query in table format. |
| **ResultSetHelper** | Represents an extended JTable, containing a table model based on a ResultSet and the corresponding query. |
| **ResultSetTable** | Represents an extended JTable, containing a table model based on a ResultSet and the corresponding query. |
| **ResultSetTableCellRenderer** | Handles the background colors for missing values differently than the DefaultTableCellRenderer. |
| **ResultSetTableModel** | The model for an SQL ResultSet. |
| **SqlViewer** | Represents a little tool for querying SQL databases. |
| **SqlViewerDialog** | A little dialog containing the SqlViewer. |

---


|  |  |  |
| --- | --- | --- |
| **Overview** \| **Package** \| Class \| **Tree** \| **Deprecated** \| **Index** \| **Help** \| **Weka's home** | |  |
| **PREV PACKAGE**   **NEXT PACKAGE** | **FRAMES**    **NO FRAMES**     **All Classes** |  |


---
